# From instinct to evidence: the role of data in country decision-making in Chile

**DOI:** 10.3402/gha.v9.32611

**Published:** 2017-05-22

**Authors:** Ximena Paz Aguilera, Consuelo Espinosa-Marty, Carla Castillo-Laborde, Claudia Gonzalez

**Affiliations:** a Centro de Epidemiología y Políticas de Salud, Facultad de Medicina, Clínica Alemana Universidad del Desarrollo, Santiago, Chile; b Instituto de Estudios Avanzados, Universidad de Santiago de Chile, Santiago, Chile

**Keywords:** Health system, health reform, health care, AUGE, priority setting

## Abstract

**Background**: The Chilean health system has undergone profound reforms since 1990, while going through political upheaval and facing demographic, health, and economic transformations. The full information requirements to develop an evidence-informed process implied the best possible use of the available data, as well as efforts to improve information systems.

**Objective**: To examine, from a historical perspective, the use of evidence during the health sector reforms undertaken in Chile from 1990 to date, and to identify the factors that have both determined improvements in the data and facilitated their use.

**Methods**: A qualitative methodological approach was followed to review the Chilean experience with data on decision-making. We use as the primary source our first-hand experience as officials of the Ministry of Health (MOH) and the Ministry of Finance before and during the reform period considered. A literature review was also conducted, using documents from official sources, historical accounts, books, policy reports, and articles published in indexed journals reviewing and discussing the reform process, looking for the use of data.

**Results**: The Chilean health-care reform process was intensive in its use and production of information. The MOH conducted several studies on the burden of disease, efficacy of interventions, cost-effectiveness, out-of-pocket payments, fiscal impact, social preferences, and other factors. Policy and prioritization frameworks developed by international agencies strongly influenced the use of data and the study’s agenda.

**Conclusions**: The Chilean example provides evidence that tradition, receptiveness to foreign ideas, and benchmarking with international data determined the use of data, facilitated by the political influence of physicians and, later, other technocrats. Internationally comparable statistics are also shown to play a significant role in the policy debate.

## Background

This article is a case study of the development in Chile of a culture of data use and evidence-informed policy-making over several decades, during which Chile went through many major political upheavals and faced demographic, health, and economic transformations. The article examines the use of evidence during the health sector reforms undertaken in Chile from 1990 to date. It analyses its context, from a historical perspective, and attempts to identify the factors that have both determined national data improvement and facilitated their use. The Chilean example provides evidence that tradition, receptiveness to foreign ideas, and benchmarking with international data shaped the use of data, assisted by the political influence of physicians and, later, other technocrats. Except for during the Pinochet dictatorship, the long-term political driver in decision-making has been the pursuit of equality.

This article covers six main topics. The first four sections focus on the drivers and uses of national data and national estimates. The second part, with two sections, discusses first the use of domestic and global estimates, and secondly, how to build capacities for data collection, analysis, and interpretation for policy-making.

## Data and methods

This article uses a qualitative methodological approach to undertake a case study of the Chilean experience with data on decision-making. As primary sources of information, we use our first-hand experience as officials of the Ministry of Health (MOH) and the Ministry of Finance during the 2005 health reform policy formulation and later discussion in Congress, and before since 1990.

Secondly, the methodological strategy included a literature review, considering official sources from the MOH, the World Bank, and the Chilean National Library of Congress, as well as several books, historical accounts, and policy reports documenting health policy-making in Chile during the twentieth century. The review was complemented by a systematic MEDLINE search using the terms ‘Health Care Reform [MESH] and Chile [MESH]’ and ‘Chile [MESH] and AUGE [all fields]’ (72 and 15 articles, respectively). From the resulting items, only those describing health reforms were finally considered, looking specifically for the use of data.

### The health system in Chile: context

Chile is a South American country with a population of nearly 18 million people, 81 years of life expectancy at birth, and high per-capita income, but where inequality remains one of the greatest challenges [1–3]. Health insurance coverage is high (98%), but structural segmentation results in low-income and high-risk populations being treated mainly in an overloaded and underfunded public system, based on social justice, while the private system treats high-income and low-risk populations, based on market justice. The two systems differ significantly in available resources, services utilization, and user satisfaction [3–5].

Although total health expenditure has increased, Chile has one of the lowest figures for total health spending among Organisation for Economic Co-operation and Development (OECD) countries. It has a low proportion of public expenditure on health (<50% of total health expenditure) and is second only to the USA regarding the share of private financing, mainly the result of out-of-pocket payment by individuals. According to the OECD, health financing in Chile ‘remains inefficient and inequitable’ [6,7].

### A short history of political drivers of better data for decision-making

Chile has a long tradition of collecting and publishing national statistics. In 1843, the statistical service was established, and in the same year the population censuses law was enacted [8]. In the context of the Latin American region, there was an early organization of both health system and social security. Key milestones in the history of social security were the creation of mutual aid societies in the mid-nineteenth century, the organization of a National Public Welfare Commission in 1877, a national coordination of charities, and the enactment of the Workers’ Compulsory Insurance Fund Law in 1924 [9,10].

It has been suggested that European liberal ideas greatly influenced the development of a medical culture with a deep interest in social problems, which furthered social policies in health. Indeed, liberal trade and openness during the war of independence allowed the unrestricted arrival of many European physicians, especially from the UK and France. They had significant professional and social prestige, and influenced the creation of the medical school, with a positivist approach based on evidence and scientific rigour. Some of them also developed successful political careers [11,12]. In the late nineteenth and early twentieth centuries, several physicians served as congressional representatives or were cabinet ministers in different executive branches (education and justice, industry, war and, later, health). They combined a scientific, data-driven approach with a strong policy commitment to change. They were instrumental in the creation of the Hygiene Institute (1892), the enactment of the Sanitary Code (1918), the Workers’ Compulsory Insurance Fund Law (1924), and the creation of the MOH (1924) (11,13).

An early example of health policies based on a scientific approach is the enactment of the Mother–Son Law in 1936, which aimed to reduce infant mortality and prevent the causes of disease and premature disability. The Minister of Health, Cruz-Coke, was a prominent physician, a scholar, and a leading researcher. At the beginning of his administration, he commissioned several studies to support effective evidence-based policies. He also enacted the Preventive Medicine Law in 1938, which enforced periodic screening tests to detect and provide prompt treatment to priority health problems, such as tuberculosis (TB) and syphilis. His surveys and data analysis provided evidence of the burden of avoidable morbidity and mortality among the working class, due to the lack of access to health care. Cruz-Coke was elected to the Senate (the upper house of parliament) for the Conservative Party after his period in the Ministry. He unsuccessfully ran for president in 1946, remaining in the Senate for several years [14–17].

The single most significant development was Salvador Allende´s book ‘La Realidad Médico-Social Chilena’ (The Chilean Socio-Medical Reality, 1939), which offers an outstanding example of the use of national data, along with global comparative analysis, to underpin a health policy proposal. The book was a real manifesto of social epidemiology, and is considered the starting point of the Social Medicine movement in Latin America [18,19].

Allende, Minister of Health at the time, describes the situation of a country in which 73% of the population lived in poverty. The infant mortality rate was 250 per 1000 live births, almost the highest recorded in the world at that time, and only half of the children born each year would live beyond the age of 10 years. This was very different from what we see today, where the infant mortality rate is slightly over seven per 1000 live births. Chilean society showed marked structural inequalities, which were exacerbated by the 1939 Chilean earthquake [18].

According to Waitzkin, in his book Allende conceptualized illness as a disturbance of the individual fostered by social deprivation, a vision deeply influenced by Virchow´s views about the social determinants of health [19]. Allende defines and analyses the priority health problems based on mortality statistics, highlighting maternal and infant mortality, TB, venereal diseases, emotional disturbances, and occupational illnesses. Compared with other countries, in 1939 the risk of premature death among Chileans was at least double that for Uruguayans, Argentines, and Colombians. National data provided a picture of the living conditions of the working classes that generated high levels of mortality and morbidity. Allende concluded the book with the MOH’s proposals for health improvement, which emphasized social change rather than medical interventions, such as income redistribution, a national housing programme, and industrial reforms, made viable through land reform and the nationalization of natural resources, among other measures [18,20–22].

The consequences of the earthquake contributed to the sense of urgency around the need to unify and reform the health and social security systems created in previous decades. Then, in 1941, Allende sent a bill to Congress to implement a health system based on socialized medicine. Ten years later, the law was enacted, with significant changes, creating the National Health Service (NHS), the first in the Americas to guarantee universal health care (Law 10,383 of 1952) [20,23].

The NHS, which operated through a countrywide network of services, had a profound impact on health indicators, with a sharp decline in TB mortality and a progressive improvement of maternal and child indicators [10]. However, during the period of military dictatorship (1973–1989), the NHS was restructured following neoliberal principles that sought to reduce the role of public institutions and to create competition both in health care and in the health insurance market [24,25].

### Contemporary use of data for economic, social, and health decision-making

The restoration of democracy in 1990, with the promotion of social justice with macroeconomic balance and growth as centrepieces of the Chilean government’s development agenda, brought new impetus to the development of social statistics and inequality analysis to underpin policy-making in health [26].

During the dictatorship, health expenditure was sharply reduced, with no investment in hospital infrastructure for 17 years. Primary-care level expansion was maintained, however, devoted mainly to maternal and child health problems. A comprehensive national health information system existed, collecting demographic, epidemiological, and health resource utilization data. Nevertheless, there was little practical use of this data for health situation analysis or health needs assessments, while financial analysis was the primary planning activity. Conversely, owing to the political emphasis on efficiency and targeting to alleviate extreme poverty, new instruments were developed, such as the Socio-Economic Characterization Survey (known by its acronym CASEN) to measure poverty and allocate social aid, including health care [14,24].

By 1990, the health system was in a deep crisis caused not only by a lack of resources, but also by inefficiency in service delivery, related to distortions of health financing mechanisms. The lack of complementarity among different levels of care was a critical problem, as was the inadequate response to changing health needs associated with the demographic transition. Government policies favoured primary health care and the private sector, neglecting the modernization and maintenance of public hospitals. Cardiovascular diseases had been the leading cause of death since 1970, but the priority in spending and health-care resources went to maternal and child health and communicable diseases; an enormous unmet demand for specialist care and tertiary hospitalizations existed [26,27].

This was the context within which in 1990 the Chilean government initiated a health reform, with financial and technical assistance from the World Bank, aiming to improve the effectiveness, efficiency, and quality of health services, particularly for poor people. Economists specializing in social policies provided the technical leadership for the reform, which involved numerous young professionals: economists, engineers, lawyers, and physicians. Many of them later took up important positions in the government and international organizations; a generation of technocrats.

The preparatory phase consisted of several studies, covering all components and entities of the health system. The findings of these studies led to the reorganization of the MOH and transformation of payment methods, from fee for service to prospective per-capita and diagnosis-related payment in primary health care and hospitals, respectively. In parallel, investments in health facilities for a new health-care model were implemented, including new ambulatory facilities, designed to deliver secondary and tertiary services [26,28].

However, the reform maintained the structural design inherited from the Pinochet dictatorship. As a result of the introduction of market justice in social health insurance during the 1980s, the health system became highly segmented, a strong determinant of inequality in access to health care. The incoming government had other significant challenges in the early 1990s, to restore democracy and demonstrate good governance. There simply was no political ground for structural changes in the health sector, and there has not been so far.

### Influence of burden of disease estimates on national policy setting

In 1994, the World Bank published its report ‘Chile: the adult health policy challenge’, which analysed the implications of demographic and epidemiological trends for health-care expenditure and intervention programmes. The first ever estimates of the Chilean burden of diseases and injuries, measured in disability-adjusted life-years (DALYs), were produced, based on simulations of national mortality data. These provided the basis for the definition of health priorities and health technology assessment [29].

In the previous year, the World Bank had published its controversial ‘World Development Report 1993: Investing in health’, which proposed packages of public health measures and essential clinical care priorities for health spending, based on the estimation of the burden of disease and the cost-effectiveness of interventions. Influenced by these ideas, the Minister of Health – an economist – decided to carry out a national estimation of DALYs based on national empirical data rather than the earlier statistical models used by the World Bank. This was the third such study in Latin America, after Mexico and Colombia. Chilean professionals from the MOH executed the study, with direct technical assistance from Harvard University. A study of social preferences in health, using qualitative research methods, was conducted in parallel by the MOH. Both studies were designed to support decision-making for prioritizing investments in health [30,31].

Since their introduction, there have been many criticisms of the use of DALYs, partly because of their complexity, but also owing to ethical problems and controversies around the use of parameters such as the differential societal value of individuals at different ages, the disability weights, and the discount rate [32]. This controversy was no different in Chile, where there was opposition to the potential use of DALYs to design basic packages and demand subsidies.

However, DALYs were instrumental in emphasizing the importance of leading causes of disability, such as depression, substance abuse, and musculoskeletal disorders. Chileans were conscious of the relevance of these health needs in a rapidly ageing population, as shown in health perception studies, and this helped to validate the use of the indicator.

**Figure 1.**
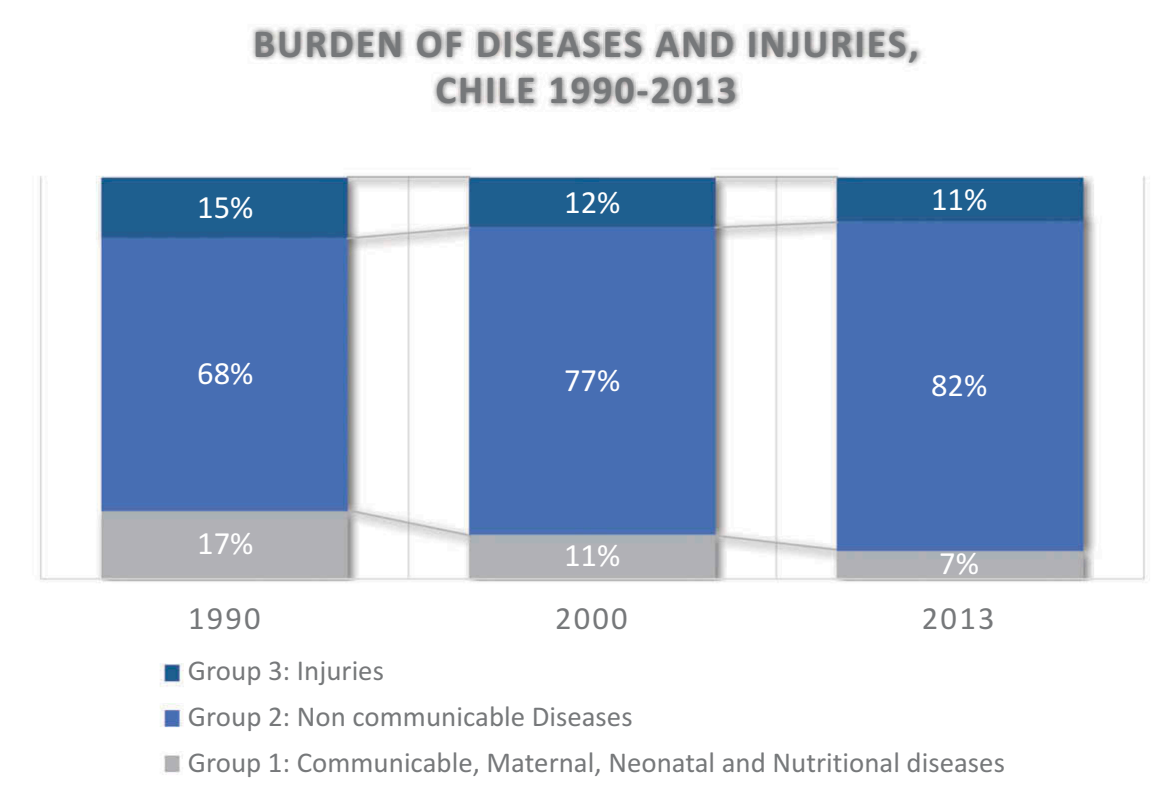
Burden of diseases and injuries in Chile, 1990–2013.

**Figure 2.**
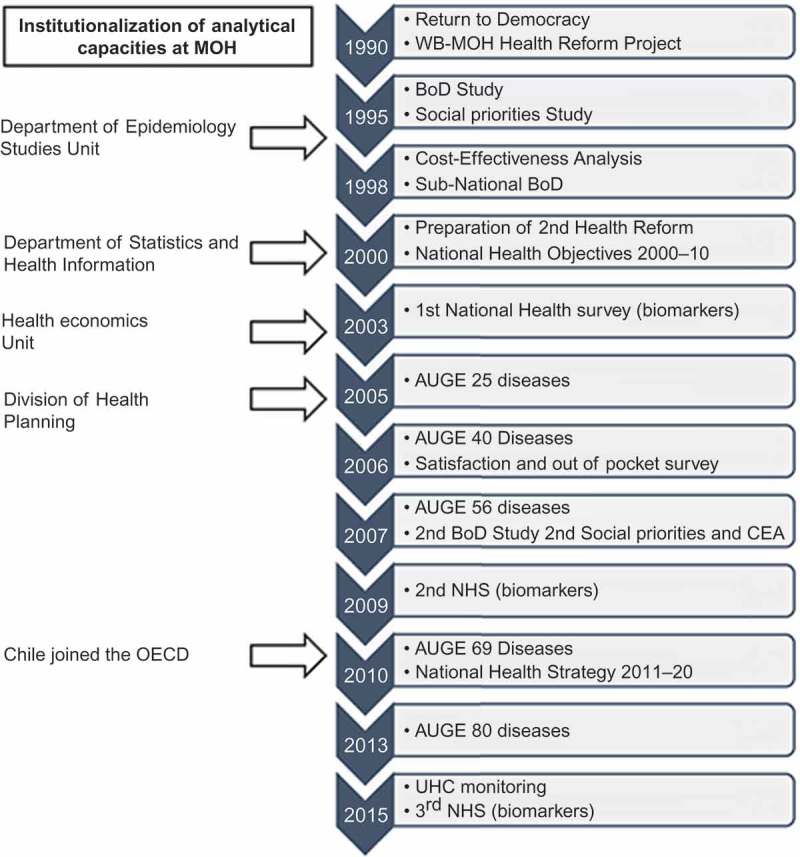
Proposed model for the prioritization of health interventions.

The results of the burden of diseases study had a profound impact on reorienting health policies (Figure 1). The study measured the mental health burden for the first time, as well as other chronic conditions, and led to plans to address them [33]. Subsequently, the MOH performed the first study on the cost-effectiveness of health interventions, using cost per DALY averted. These initiatives were instrumental in institutionalizing epidemiological analysis capacities within the MOH and defining an agenda for strengthening public health surveillance. Using both studies, the MOH proposed a first model for prioritizing health interventions, adapted from Bobadilla´s work, which would later be used to define health priorities for the new health reform in 2000 (Figure 2) [34].

Health inequality between population groups and inequality in access to health care were the focus of attention during this period. In 1998, the MOH, together with the Pan American Health Organization, convened an international seminar on ‘Equity and efficiency in decision-making in health’, fostering broader discussion on these issues. The main topics were the impact of the social determinants of health, geographical inequality in the burden of diseases, and the mismatch of the health system to the priority needs. The burden of disease demonstrates the preponderance of non-communicable diseases, and despite increased investment during the 1990s, the public health system still lacked the resources to address the new health priorities. Therefore, the private–public gap remained a significant equality concern [35,36].

The discussion also covered MOH strategies to address inequality in access, including the new public insurance (FONASA) prioritizing the benefits mechanism, such as the ‘program of complex services’, based primarily on cost and waiting lists, instead of epidemiological criteria.

### Extensive role of country data in the Regime of Explicit Health Guarantees (AUGE)

In 2000, a new cycle of health reforms began in an attempt to reduce inequalities. The Chilean government implemented an innovative second-generation health reform, the central focus of which was the recognition of the right to health. A physician headed the Inter-ministerial Committee leading the process, which involved the Ministries of Health and Finance, and the General Secretariat of the Presidency [37]. On this occasion, the reform was designed without technical or financial support from donor agencies, and included a new legal framework for the health system. It made extensive use of data and national studies.

**Figure 3.**
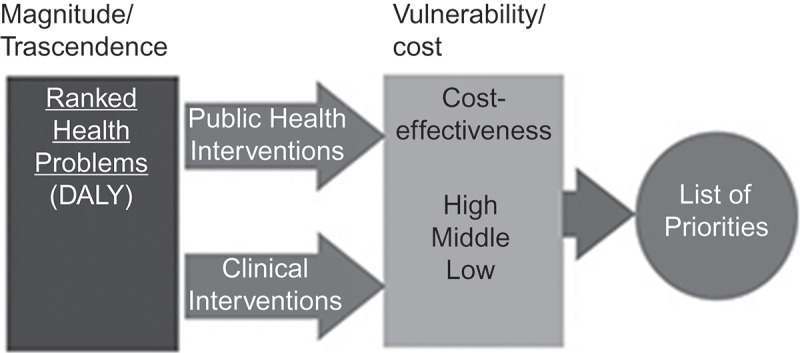
Four health objectives for the decade 2000–2010.

The first step was the definition of health objectives for the decade 2000–2010, called ‘Objetivos Sanitarios para la Década 2000–2010’ (OS2000-10) (Figure 3). OS2000-10 provided strategic direction and set the rules for all the different actors involved in health reform, as well as establishing a baseline to evaluate the impact of the reform [38]. Two aspects are worthy of particular attention. The first was the inclusion of an objective on people’s expectations of the health system (out-of-pocket payments, responsiveness, and quality of services), influenced by the World Health Report 2000 conceptual framework. The second was the explicit priority setting, based on national data, which used the model developed previously, based on DALYs and cost-effectiveness. The proposal was broadly discussed and approved, during public hearings, by several professional and non-governmental organizations, including representatives of the health industry [38].

The primary instrument for achieving health goals was the creation of a regime of explicit health guarantees (known by the acronym AUGE) for prioritized health problems (law 19.966, 2004) [39]. The law mandates coverage by public and private insurers for the diagnosis, treatment, and follow-up of 80 health problems, mostly the leading causes of burden of diseases and injuries in Chile. To operationalize this mandate, the law sets four guarantees: (1) access (who is eligible?); (2) timely access (for eligible beneficiaries the law establishes maximum waiting times); (3) financial protection (it establishes maximum co-payments and deductibles); and 4) quality (accreditation of facilities and professional certification). It was the first example in Latin America of a rights-based social guarantee that provides operational definitions of health-care entitlements [40].

A legal decree, signed by both the Minister of Health and the Minister of Finance, defines for each of the 80 problems the requisites of eligibility, the maximum waiting times, the list of health interventions covered, and the co-payment limit. It is the responsibility of the medical insurance provider, either public or private, to comply with these guarantees. This establishes the minimum effective health coverage for every individual, regardless of their level of contribution. This feature is considered one of the main achievements of the reform [4,27,39].

Before the reform, the Chilean Constitution only stated second-generation rights regarding health care. The jurisprudence considers these as a mere statement of intention, and therefore the state and the courts are not obliged to enforce them, except for the right to choose the health system, either public or private. Since the reforms, AUGE has constituted the core of the constitutional right to health, which may be enforceable in compliance with both the AUGE law and the constitutional mandate [40–42].

AUGE was progressively implemented, beginning with 25 health problems in 2005 and reaching 80 in 2013. Priority setting used three criteria: (1) priority diseases, as defined in the health objectives of the decade; (2) the existing interventions already covered by the public insurance through the ‘program of complex services’; and (3) social preferences, including the use of the ‘rule of rescue’, the imperative that people feel to help identifiable individuals facing avoidable death [43,44]. The high burden of disease and social preferences captured 88% of the selected conditions for AUGE [45].

**Figure 4.**
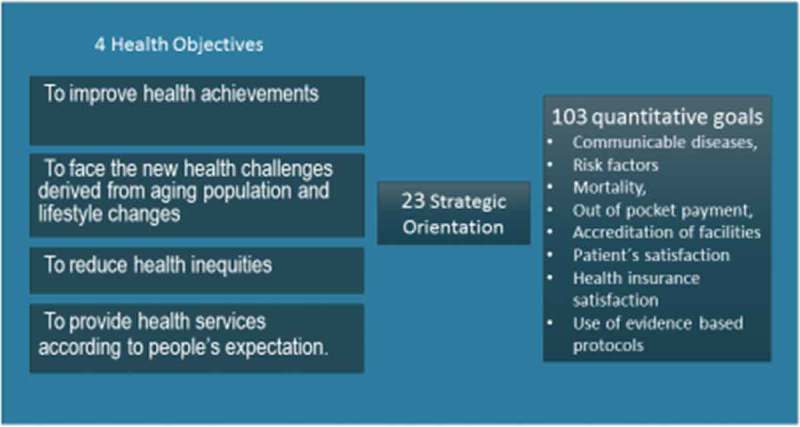
The Regime of Explicit Health Guarantees (AUGE) regime: an evidence-based approach to expand the package every 3 years.

The AUGE law sets a schedule of mandatory technical, epidemiological, and economic studies that support decision-making for updating the priority list every 3 years through a legal decree (Figure 4). The preparation of the regime also requires developing clinical guidelines for each health condition, to standardize diagnosis and treatment, with the support of systematic reviews of evidence and the involvement of scientific societies (Box 1). In this phase, the maximum waiting times for care (opportunity) are also defined.

**Box 1.** Procedure for defining guarantees (GES Regulation, Decree No. 121, 2005).

AUGE law 19.966 defines the procedure for the prioritization of problems to be incorporated into the regime of explicit guarantees. The complete cycle considers the following steps:
(1) Definition of the budgetary framework by the Ministry of Finance.
(2) Technical health and economic studies to prepare a list of health problems with associated effective interventions (diagnosis, treatment, or rehabilitation). These include burden of disease, social preferences, efficacy of interventions, and cost-effectiveness of intervention studies.
(3) Prioritization of health problem, using the following criteria: magnitude of the problem, importance of the problem (qualitative), effectiveness of Interventions, and feasibility of Implementation of Interventions. The final criterion considers cost and supply capacity in the health system. Also mentioned is the financial burden on households.
(4) Determination of the average expected cost, which requires the measurement of both the cost to deliver each intervention, and potential demand. The maximum co-payments for users of public and private insurance are also defined in this phase (financial protection).
(5) External verification of the cost by an external study, to ensure that the proposal does not exceed the budgetary framework defined in step 1.
(6) Consultation by an advisory council, comprised of independent professionals representing the faculties of medicine, economics, chemistry and pharmacy, the Chilean Academy of Medicine, and representatives of the president. This checks the consistency of the proposal.
(7) Final drafting of the decree and its enactment.

New epidemiological and statistical tools were developed to monitor the outcome and impact of the reform, which strengthened the public health surveillance system. To begin, the MOH implemented the AUGE information system, which oversees the compliance with the four legal guarantees. In addition, a national health survey, with biomarkers, was implemented, along with two other surveys on quality of life and patient satisfaction and health spending. Lastly, health questions of the CASEN household survey were adapted to measure access to and utilization of health services. Thereby, it is now possible to disaggregate access according to age, gender, ethnic origin, socio-economic status, health insurance, residence, and migrant status. It also has questions about AUGE use and perception.

Even before its enactment, the AUGE law was the target of severe criticism. One of the earliest and more relevant reproaches was the risk of neglect of non-priority health problems. The waiting lists for these have worsened, despite interventions to reduce them [46,47].

The entitlement to timely access obliges health insurers that exceed the maximum waiting time to purchase health interventions outside their network. In practice, FONASA (the public insurer) has been unable to fully comply, owing to shortages of specialists, hospital beds, and technology in public facilities. Thus, the public sector has been increasingly purchasing services from private providers, leading to rising costs and reinforcing the underfunding of public health providers.

Clearly, AUGE has been unable to overcome the problems generated by a fragmented system, segmented by income and health status. During the parliamentary discussion of the bill, right-wing parties rejected the setting up of a compensation fund to finance AUGE, designed to pool risks and create solidarity between the public and private sectors [48].

**Figure 5.**
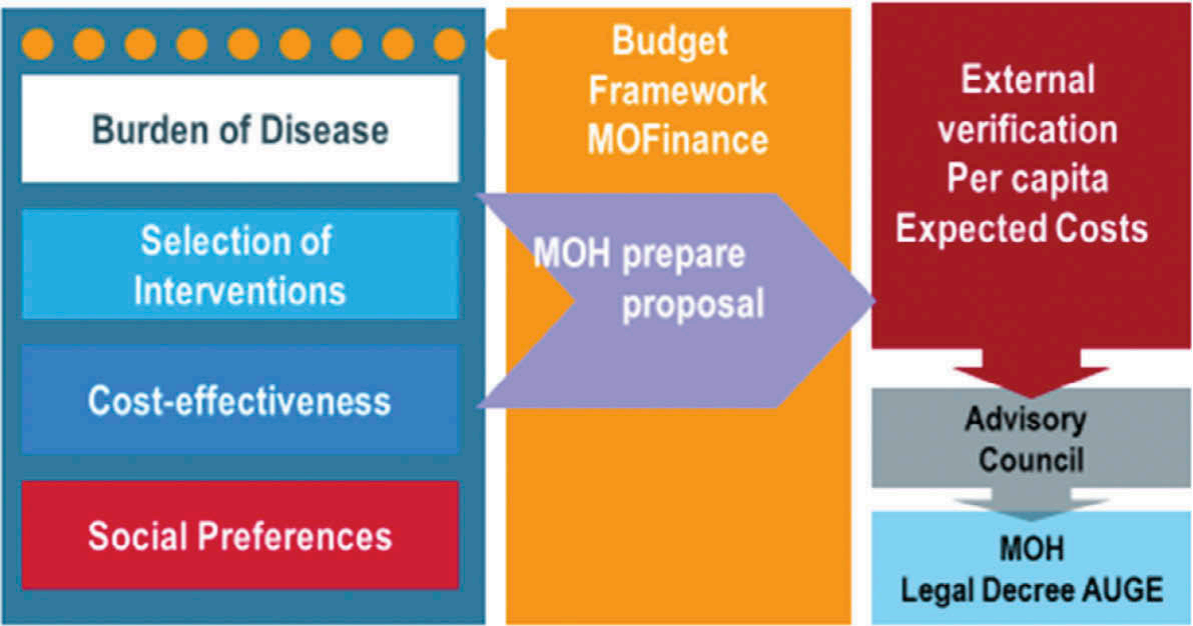
Survival after acute myocardial infarction (AMI) in Chile: comparison before and after implementation of the Regime of Explicit Health Guarantees (AUGE).

On the other hand, in the 10 years since its implementation, the reform has shown benefits regarding access and health impact. Chilean´s utilization of health-care services has increased, although gaps between the richest and the poorer people remain [49]. For example, expanded access and new protocols have improved the treatment of acute myocardial infarction in public hospitals, accelerating the decline in mortality (Figure 5) [50]. There is also evidence of increased coverage and mortality reduction for diseases such as hypertension, diabetes, stroke, and cervical and gallbladder cancer [46,51]. Finally, but perhaps most importantly, Chileans have valued AUGE highly in every study and opinion polls since its implementation.

## Discussion: uses of global and national estimates

In Chile, national data are used for decision-making and health policy design, while data and global estimates are used for comparative purposes. They serve to benchmark progress and identify areas with potential for improvement. In the absence of national data, it is customary to use global estimates, such as in the case of rare diseases, or for health intervention efficacy analysis, which is usually performed based on international data.

The World Health Organization (WHO) traditionally considers Chile among the countries with reliable statistics [52,53]. Discrepancies between national figures and estimates produced by United Nations agencies for the health sector have occurred only rarely, and have then been solved by providing credible national evidence. For example, at the end of the decade 2000–2010, the WHO estimated that the prevalence of smoking in adult Chileans was higher in women than in men of the same age. The country held the opposite view, based on the results of the national health survey. Finally, it was shown that different surveys consistently supported the position of the country, which was accepted by the WHO.

In 2010, Chile joined the OECD. This has involved a challenge for the national statistical systems, which have had to adapt to the measurements and standards of this organization. In practice, the accession to the OECD has meant changing the tradition of benchmarking progress against other Latin American countries. Thus, Chile, which had long been used to its position as the best performing country for health in the region, suddenly found itself classified among the worst performers among members of the OECD [5,7,54].

This new performance benchmarking has fuelled debate in the academic and political environment, and shows the potential impact of comparative cross-country benchmarking based on sound empirical data.

Among the main challenges for the future is the need to strengthen the health information infrastructure to improve the opportunity of data, to incorporate quality of care indicators, and to provide disaggregation to monitor equality in health coverage. The different surveys allow us to disaggregate data by gender, socio-economic and minority group status, but this is not the case for administrative registries. In a study on universal health coverage (UHC) in Chile, we described the shortcomings of the MOH registries, which impede to assess equality in health coverage for many public health programmes, such as vaccination, TB, HIV/AIDS, antenatal care, and birth control [4].

It is also relevant to update studies on the national and subnational burden of diseases, cost-effectiveness, and social preferences, to bring AUGE coverage up to date. There is an ample window of opportunity to improve health system performance through the use of both national data and international benchmarking.

## Conclusion: how to build capacities for data collection, analysis, and interpretation for policy-making

The struggle to adapt health services to changing health needs is one of the primary challenges for many low- and middle-income countries. During the past decade, Chile has opted for a data-driven priority setting and rights-based approach in health reforms, providing entitled access to health care for priority diseases. In support of this process, the MOH strengthened its information systems and created new tools to monitor results, clearing a path for other countries to follow. However, it did not change the structural conditions of the segmented health system, which is a common situation in many Latin American countries. The Chilean example has shown some gains in health status, but a persistent inequality in access to health care. It is also an example of what Schuftan has described as ‘being sucked into dreaming that technical solutions in public health would right the wrongs’ [55]. Nevertheless, it proves that it is possible and worthwhile to allocate scarce resources to the most critical health needs and cost-effective interventions.

Some factors associated with using data in decision-making persistently appear throughout history. Among them are the openness to foreign influences, first derived from the arrival of European doctors with liberal ideals, and then the international comparative analysis as an element of judgement about the national situation. It is also important to acknowledge the importance of policy and prioritization frameworks developed by international agencies.

In Chile, doctors have been influential political players in making health policy decisions, taking a positivist, data-based approach, which is currently adopted by economists. The definition of inter-ministerial working teams, addressing health problems from health, economic, and political perspectives, has helped to maintain a tradition of making decisions based on the use of data in the health sector. As the backbone of policy development, there is an ongoing need to confront structural inequalities, a feature of Chilean society that we have failed to remedy [56,57].

**Figure 6.**
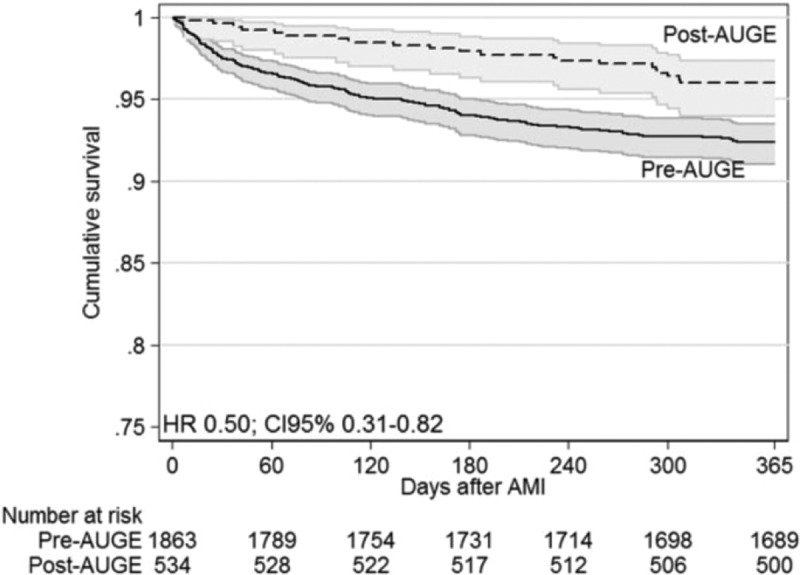
Role of data in decision-making in Chile: from 1990s’ health reforms to the Regime of Explicit Health Guarantees (AUGE).

The use of studies and national data starting during the 1990s and in the reform of 2005 give an impression of continuity in the line of thought, but in practice, the systematic adoption of evidence-informed policy and prioritization proceeded patchily (Figure 6).

There was no planned connection between the 1990s’ studies and the health reform that began in 2000 and resulted in the AUGE law. The authorities belonged to the same political coalition, but with different perspectives about the health sector.

We maintain that the studies performed during the 1990s, on the burden of diseases and cost-effectiveness of health interventions, were used because some technical teams that worked on them stayed at MOH during both periods. As already mentioned, the studies were not intended to implement a system of enforceable rights for priority diseases; they were carried out to support investment decisions on health-care infrastructure and medical equipment. However, the health authorities responsible for the 2005 reform saw that those studies could give them a technical substrate for prioritization.

Having human resources capable of generating and analysing information is certainly a necessary condition for the use of data in decision-making, but it is not a sufficient one. The authorities can make decisions with or without data, so the technical experts and statistical analysts have the responsibility to provide the best information available in a timely and understandable manner. There is a liaison between technical knowledge and the political authorities, which requires physical closeness between the two worlds. For example, the institutionalization of analytical capacity at the MOH, first in the department of epidemiology and later with the creation of the department of statistics and health economics, gave rise to the division of health planning. This division is responsible for formulating the national health strategy and the development of the necessary studies for AUGE.

It is also important to have budgets to support operational research aimed at solving specific problems of the health system, which enables the creation of links between the worlds of academia and public health management.

The Chilean experience shows that the availability of technical expertise in the MOH facilitated the use of national data in decision-making processes of the reforms from the 1990s onwards. However, the maintenance of such capacities can never be taken for granted and requires constant vigilance. Job instability and career shortcomings discourage the retention of professionals in the public service, and also threaten the political independence of the health information system.

Currently, the directors of both the National Statistical Institute and the Civil Registry and Identification Service are recruited through an open and competitive process, carried out by consultants and conducted by the public Senior Executive Service System (SESS). In contrast, directors of the health statistics and epidemiology departments at MOH are appointed by the authority, and only a minority of civil servants at MOH have tenure. During recent shifts of government, all of the directors mentioned above were replaced, despite their connection with SESS. The turnover of staff members coincided with serious problems in the quality of data, such as the failure of the last population census (2012), polemics about poverty measurements, and health population surveys being suspended or delayed [58,59].

Despite these problems, the country has maintained the discipline of decennial health plans; the current one is the national health strategy 2011–2020 [60]. Also, recent initiatives such as the law for high-cost diseases have defined a funded mechanism based on the best evidence to support decisions [61]. One area that requires further development is the need to increase the use of data and evidence in the management of health-care services, mainly focusing on studies and proposals on payment mechanisms.

Finally, global estimates and global commitments such as UHC and sustainable development goals play a major role in mobilizing the political will of the leaders to invest in their information systems and generate evidence for decision-making. Transparency in the handling of data by agencies such as the WHO and accountability of international commitments are strong incentives for resource mobilization for capacity building in the capture, analysis, and use of data.

Chile’s experience with international estimates, particularly in the 1990s, shows how these can have an influence within a country, even though national estimates take precedence in local decision-making. The global progress in methods to provide comprehensive estimates of the burden of disease has led to advances in analyses in Chile, with international technical support, and influences the national analytical work, including estimates. In addition, internationally comparable statistics are a valuable input into the policy debate.

## Paper context

Health systems in Latin America have struggled to adapt to changing health needs caused by rapid demographic transition and recent economic growth. The new health priorities non-communicable diseases, addictions, and violence are added to the maternal and child health and infectious problems, still not fully resolved. Health reforms in Chile, since the 90s to date, provide lessons from a sustained effort to adapt, based on data and scientific grounds, with lights and shadows.
